# Arrhythmia in Acute Myocardial Infarction: A Six-Month Retrospective Analysis From the National Institute of Cardiovascular Diseases

**DOI:** 10.7759/cureus.11322

**Published:** 2020-11-04

**Authors:** Abdul Mueed, Shahzad Khatti, Jibran Ashraf, Khawaja M Aarij, Muhammad Waqas, Tariq M Khan

**Affiliations:** 1 Cardiac Electrocardiography, National Institute of Cardiovascular Diseases, Karachi, PAK; 2 Interventional Cardiology, National Institute of Cardiovascular Diseases, Karachi, PAK; 3 Cardiology, National Institute of Cardiovascular Diseases, Karachi, PAK; 4 Noninvasive Imaging, National Institute of Cardiovascular Diseases, Karachi, PAK; 5 Cardiac Surgery, College of Physicians and Surgeons Pakistan, Karachi, PAK

**Keywords:** arrhythmia, acute myocardial infarcation, non-st segment elevation myocardial infarction (nstemi), st-elevation myocardial infarction (stemi), av nodal block, ventricular tachycardia (vt), bradycardia, atrial fibrillation

## Abstract

Introduction

Acute myocardial infarction (AMI) is a devastating medical emergency that requires immediate pharmacological and radiological intervention. With the advent of techniques such as percutaneous coronary intervention (PCI), pacemakers, and percussion pacing, survival rates have improved significantly. However, there are certain factors and complications associated with AMI that still lead to a high mortality rate, such as old age, advanced heart disease, diabetes mellitus (DM), and arrhythmias.

Factors such as the type of arrhythmia, the heart rate, and the level at which dissociation occurs between atrial and ventricular rhythm all influence mortality and morbidity rates. Outcomes are further influenced by the sex of the patient, the type of AMI [ST-elevation myocardial infarction (STEMI) or non-ST-elevation myocardial infarction (NSTEMI)], history of smoking, arrival times at the hospital, presence of hyperglycemia, previous history of cardiac surgery, and the need for a temporary pacemaker or a permanent pacemaker.

As with most scientific studies, local data from Pakistan is hard to find on this topic as well. With this study, we hope to contribute valuable information and updates to the study of a developing problem from the developing world.

Objective

We aimed to analyze the frequency and outcomes of different types of arrhythmia in AMI.

Methods

This study involved a retrospective observational cohort. It was conducted at the National Institute of Cardiovascular Diseases (NICVD), Karachi from January 2019 to July 2019 (six months). All data were retrieved from the online database at the NICVD. Written consent was obtained from all patients. Patient confidentiality was ensured at all times.

Results

A total of 500 patients were included in the study. The mean age of our cohort was 56.17 ±14.01 years. NSTEMI was more prevalent than STEMI. Sinus arrhythmia (SA) was the most frequently recorded arrhythmia and had the best survival rates. Atrioventricular (AV) nodal blocks and ventricular tachycardia (VT) had the worst outcomes. The overall mortality rate was 11.4%, and the mean in-hospital length of stay was 2.07 ±1.54 days. Smoking increased mortality in all cases.

Conclusions

AMI is complicated by several types of arrhythmia. SA is the most common arrhythmia in AMI. Mortality in AMI is largely due to AV nodal blocks and VT. Smoking increases mortality in all cases.

## Introduction

Cardiovascular disease (CVDs) impart the greatest burden to healthcare systems around the world; not only are CVDs costing more and more to treat but their incidence is on the rise due to our modern-day sedentary lifestyle [1]. Ischemic heart diseases (IHDs) including myocardial infarction (MI) are the leading causes of CVD-related deaths globally, accounting for nearly nine million deaths annually [2]. IHDs are usually complicated by cerebrovascular diseases, hypertension (HTN), diabetes, smoking and other types of tobacco use, conduction defects, and other comorbidities, which all contribute to an increase in morbidity and mortality [3].

Acute myocardial infarction (AMI) represents the most life-threatening manifestation or complication of IHD. Almost all AMIs require emergency intervention, be it pharmacological or radiological. With early detection and intervention via evolving technologies such as percutaneous coronary intervention (PCI) or temporary pacemakers, survival rates for AMI have drastically improved [4].

However, despite optimal management and development of groundbreaking techniques, the mortality rates for AMI have remained steady over the last decade [5]. Mortality rates may have reached a plateau, but some aspects or complications of AMI can still generate significant morbidity and mortality, perhaps contributing to the plateau seen with recent data [5,6].

One such factor resulting in worse outcomes is AMI associated with arrhythmia(s); previously published data have conclusively proven that AMI associated or complicated with any arrhythmia leads to increased mortality even with early detection and intervention when compared to AMI without arrhythmia [7]. Furthermore, the type of arrhythmia and the level at which dissociation occurs between atrial and ventricular rhythm can significantly determine eventual outcomes [8].

The National Institute of Cardiovascular Diseases (NICVD), Karachi is the largest tertiary care center that specializes in cardiology in Pakistan. Despite witnessing an inordinate amount of patients every day, published data from our institute is still scarce. Our study aims to rectify this by observing and analyzing outcomes in patients presenting with AMIs complicated by arrhythmias.

## Materials and methods

This was a retrospective observational study conducted at the NICVD, Karachi from January 2019 to June 2019 (six months). Patients who were 18 years or older and admitted with a confirmed diagnosis of AMI with an associated arrhythmia were included in the study. Patients with advanced heart failure, renal failure, previous episodes of MI, multiple organ failure, and terminally ill patients were excluded from the study. Written consent was obtained from all patients. Patient confidentiality was of the highest importance and ensured at all times.

AMI was diagnosed on the basis of the adopted definition of AMI by the Joint European Society of Cardiology (ESC)/American College of Cardiology (ACC)/American Heart Association (AHA)/World Heart Federation (WHF) Task Force [9]. These guidelines have provided three definitions for AMI: ST-segment elevation definition, universal definition, and the old definition. The universal definition of MI denotes the presence of acute myocardial injury detected by abnormal cardiac biomarkers in the setting of acute myocardial ischemia; this definition is not very specific and rarely used. The old definition has now become obsolete. For the purposes of this study, we used the ST-segment elevation definition and classified the AMIs into two groups: ST-segment elevation myocardial infarction (STEMI) and non-ST segment elevation myocardial infarction (NSTEMI).

STEMI was defined as the presence of persistent ST-segment elevation in at least two contiguous leads or any equivalent [left bundle branch block de novo, or presumably de novo, or ST-segment depression in the precordial leads (V1-V3) on electrocardiogram (ECG) at admission associated with symptoms suggestive of myocardial ischemia]. NSTEMI was characterized as the absence of ECG changes found in the STEMI but with symptoms and markers suggestive of MI.

The ACC, AHA, and ESC guidelines were used for defining arrhythmias [10]. Atrial fibrillation (AF) was defined as a supraventricular tachyarrhythmia characterized by uncoordinated atrial activation with consequent deterioration of atrial mechanical function. Ventricular tachycardia (VT) was defined as a ventricular tachyarrhythmia characterized by uncoordinated atrial activation with consequent deterioration of ventricular mechanical function.

Atrioventricular (AV) block was defined as the complete or partial dissociation between atrial and ventricular rates with the atrial rate being greater than the ventricular rate and narrow escape rhythm. In patients who presented with or developed conduction defects, the site of the defect was recorded and the defect was classified as AV nodal block, first-degree AV block, second-degree AV block, third-degree AV block, or complete heart block.

A heart rate of <40 beats/minute not associated with a conduction defect and refractory to the administration of atropine was classified as bradycardia. Sinus arrhythmia (SA)was defined as a heart rate of 100 or more beats/minute with a regular rhythm along with upright P waves in leads I, II, and aVL plus negative P waves in lead aVR, and with each P wave followed by a QRS and T waves. The terms sinus tachycardia (ST) and SA are used interchangeably.

Primary endpoint

Mortality at seven days (one week) was the primary endpoint of this study. The duration of stay at the hospital was also analyzed.

Statistical analysis

Data were analyzed using SPSS Statistics, version 25.0 (IBM, Armonk, NY). Mean and standard deviations were calculated for age and duration of hospital stay. Frequencies were calculated for gender, comorbidities, mortality, types of MI, and types of arrhythmia.

## Results

The mean age of our cohort was 56.17 ±14.01 years. The male-to-female ratio was 2:1. A majority of the patients had both HTN and diabetes mellitus (DM). But, surprisingly enough, the most common risk factor for adverse cardiovascular events was smoking; nearly three in five patients had a history of smoking. Exactly two-thirds of the patients presented with an NSTEMI. General patient characteristics and types of AMI are summarized in Table 1.

**Table 1:** Demographics and cardiovascular disease-specific characteristics of the patients included in the study HTN: hypertension; DM: diabetes mellitus: AMI: acute myocardial infarction; NSTEMI: non-ST-elevation myocardial infarction; STEMI: ST-elevation myocardial infarction; SD: standard deviation

Variables	Values (N=500)
Age in years (mean ±SD)	56.17 ±14.01
Gender	
Male, n (%)	318 (63.6%)
Female, n (%)	182 (36.4%)
Comorbidities	
Smoking, n (%)	283 (56.6%)
HTN + DM, n (%)	183 (36.6%)
DM only, n (%)	92 (18.4%)
HTN only, n (%)	12 (2.4%)
AMI variants	
NSTEMI, n (%)	333 (66.6%)
STEMI, n (%)	167 (33.4%)

Seven different types of arrhythmias were encountered in our study. Half of the patients presenting with an AMI had SA. AV blocks (first, second, and third degrees) were the second most common arrhythmia recorded. The frequency of different types of arrhythmia is shown in Table 2.

**Table 2:** Frequency of different types of arrhythmia recorded in the study AV: atrioventricular

Type of arrhythmia	Frequency, n (%); (N=500)
Sinus arrhythmia	250 (50%)
First-degree AV block	88 (17.6%)
Third-degree AV block	64 (12.8%)
Atrial fibrillation	45 (9.0%)
Bradycardia	24 (4.8%)
Ventricular tachycardia	18 (36%)
Second-degree AV block	11 (2.2%)

The overall mortality rate at seven days was 11.4%, with a mean hospital stay of 2.07 ±1.54 days. AV nodal blocks were the biggest contributors to the mortality rate. The mortality rates related to the complications are presented in Table 3.

**Table 3:** Overall and relative mortality rates AV: atrioventricular; SD: standard deviation

	Mortality at one week, n (%)	Relative mortality within the type of arrhythmia (%)
Third-degree AV nodal block	24 (4.8%)	37.50%
Ventricular tachycardia	12 (2.4%)	66.66%
Bradycardia	8 (1.6%)	37.50%
Atrial fibrillation	7 (1.2%)	15.50%
Sinus arrhythmia	4 (0.8%)	1.60%
Second-degree AV nodal block	2 (0.4%)	18.18%
First-degree AV nodal block	Nil	Nil
Overall mortality	57 (11.4%)	
Length of hospital stay, days, mean ±SD	2.07 ±1.54	

## Discussion

Fabijanic et al. have previously reported a higher incidence of comorbidities and increased in-hospital mortality for female cardiac patients when compared to male cardiac patients [11]. Similar results were also recorded by Trappolini et al. [12]. Both of these studies demonstrated an increased susceptibility among females to arrhythmia(s); arrhythmia acts as an independent risk factor for increased mortality in patients with AMI. We did not reach similar results in our study. Overall mortality was quite low in our analysis, resulting in similar survival rates for men and women. Secondly, almost all smokers were men, which reduced survival in men and, conversely, the absence of smokers among women improved survival rates. Men also have easier and greater access to healthcare facilities, which might have contributed to their predominant numbers [13]; however, despite previous reports, this remains an expert opinion at best.

The prevalence of DM and HTN was in line with previous reports [14]. Remarkably, both of these risk factors were overshadowed by smokers, who made up more than half of all patients. This is very concerning as recent data has shown that in Pakistan, the most susceptible individuals take up smoking at a young age and continue with the habit even in the face of clear and present danger [15]. It stands to reason that even when the realization of the enormity of this hazard sets in, quitting smoking remains arduous and patients irrespective of their cardiac status go through multiple cycles of quitting, trying to quit, using after quitting, and trying to reduce the number of cigarettes per day [16]; our results revealed that most of them eventually do continue with the habit. 

The incidence of NSTEMI versus STEMI does not have a uniform presentation among different cohorts and populations. The incidence of STEMI from the RENASCA analysis was reported to be approximately 74% [17]; yet, Hong et al. from Japan [18] and Jurado-Román et al. from Spain [19] reported a higher incidence of NSTEMI compared to STEMI. The incidences of DM, HTN, and smoking were comparable in all three reports. The one apparent difference among the three cohorts was the mean age at presentation. Similar to our results, studies with relatively younger patients had a higher incidence of NSTEMI compared to STEMI. However, there were marked geographical variations in frequency and incidence between the two subtypes of AMI, which we have not accounted for.

Half of the patients included in the study had SA. SA per se is not a pathological finding. Its incidence in AMI has previously been reported to be at around 45%; SAs are associated with a very low mortality rate, reduced in-hospital stay, cost of treatment, and the need for revascularization [20]. Our findings validate the previously published data. In-group mortality for AMI with SA was less than 2% with age being the only significant factor adversely affecting survival rates in this subgroup.

AV nodal blocks frequently complicate AMI, but they typically occur with the STEMI variant [21]. AV nodal blocks are the most commonly acquired arrhythmia seen with idiopathic fibrosis and AMI [22]. In our study, the percentage of AV nodal blocks was relatively low; this was largely due to the low number of STEMI cases among our cohort. However, relative percentages can be misleading. It has to be noted that SAs are not pathological as discussed above and are considered a normal benign finding. Factoring this into our evaluation cuts the cohort in half. Of the remaining half, >60% of the patients had AV nodal blocks, ergo even with our limited sample size, AV nodal blocks represented the most regularly acquired arrhythmias.

First-degree AV block is not only the most prevalent among the three subtypes but also the most benign and amenable to treatment [23]. There were no mortalities in this subgroup of AV blocks. By contrast, third-degree AV block was the single largest contributor to deaths in our study. Third-degree AV blocks have a reported mortality rate of 20-30% [24], but these numbers jump up significantly in the presence of DM and smoking [24]. It is clear that the presence of a massive number of smokers and diabetics had an adverse effect on survival rates in our study. Only 11 patients with second-degree heart blocks were recorded, and this number was too low to perform any significant statistical analysis. Second-degree AV blocks can progress to a complete heart block or third-degree heart block [25], and this is our best guess as to why there were so few of these cases recorded.

Bradycardia in the setting of AMI occurs only in a minority of the patients [26]. Although occurring only in small numbers, this bradycardia nonetheless carries a significant risk of transforming into bradyasystolic cardiac arrest that is refractory to atropine or epinephrine administration. Once this bradyasystole occurs, the mortality can be as high as 44% [27]. Novel therapies such as the use of aminophylline and percussion pacing have been employed to manage this condition, but mortality rates have remained high [28], which is consistent with our outcomes. It should be noted that bradycardia was defined by its rate and when it became refractory to atropine administration (see Materials & Methods).

Unlike other arrhythmias, AF occurs in older female patients without a history of smoking. AF can either be silent or symptomatic during an AMI. The incidence of silent AF and symptomatic AF during AMI has been reported to be 5-9% and 13-16% respectively [29]; the incidence of silent AF is probably higher as their inherent nature makes the diagnosis challenging. Irrespective of the type, AF acts as an independent factor for increased in-hospital mortality and post-AMI adverse events [29]. Previously, in-hospital mortality rates of 25-45% were reported; these have since improved with the use of oral anticoagulants, and we encountered similar outcomes. 

Historically, AMI with VT has been associated with abysmal mortality rates, with rates exceeding 60% at 30 days post-infarction [30]. Male sex, hyperglycemia, and delays in the intervention are further associated with reduced survival in AMI with VT [30]. Even though VT was the least frequent arrhythmia observed in our study, it still caused the second-highest overall mortality and the highest in-group mortality; two-thirds of the patients with VT died while still at the hospital. This low survival rate was definitely influenced by the highly prevalent bad prognostic factors in our study, i.e., male sex and DM. Arrival time at the hospital was generally very short.

Limitations of the study

The authors would like to acknowledge that patient follow-ups could not be kept up for longer periods of time. This has most probably resulted in significantly high survival rates; all-case mortality is higher in previously reported data at one year or more. Dyslipidemia, metabolic syndrome, and the use of statins, antihypertensive medications, oral anticoagulants, and aspirin were neither recorded nor analyzed. Data for nutritional assessment was not available to us.

## Conclusions

AMI is associated with several types of arrhythmias. SA is the most frequent arrhythmia seen in AMI; it also has the highest survival rate. Third-degree AV block and VT have the highest mortality rates among all arrhythmias seen in AMI. All-case mortality rates are aggravated by old age, a history of smoking, and DM.
